# The Effect of Foliar Fungicide and Insecticide Application on the Contamination of Fumonisins, Moniliformin and Deoxynivalenol in Maize Used for Food Purposes

**DOI:** 10.3390/toxins14070422

**Published:** 2022-06-21

**Authors:** Massimo Blandino, Valentina Scarpino, Giulio Testa, Francesca Vanara, Amedeo Reyneri

**Affiliations:** Department of Agricultural, Forest and Food Sciences (DISAFA), Università degli Studi di Torino, Largo Braccini 2, 10095 Grugliasco, Italy; valentina.scarpino@unito.it (V.S.); giulio.testa@unito.it (G.T.); francesca.vanara@unito.it (F.V.); amedeo.reyneri@unito.it (A.R.)

**Keywords:** corn, mycotoxin, *Fusarium*, good agricultural practices, prothioconazole, tebuconazole

## Abstract

The fungal ear rot of maize cultivated in temperate areas is mainly due to the *Fusarium* species. The use of insecticides against European Corn Borer (ECB) reduces the severity of fungal ear rot as well as the fumonisin (FB) and moniliformin (MON) levels in maize kernels at harvest, which in turn results in a lowering of their effect on deoxynivalenol (DON) control. However, the direct fungicidal control of ear rot has rarely been implemented for maize, and the first studies reported conflicting results on the reduction of mycotoxins. In the present experiment, field trials were carried out in North Italy over three growing seasons to study the effect of fungicide application timings on maize to control mycotoxins, considering the interaction of the application with the insecticide treatment, according to a full factorial split plot design. The mycotoxin content was determined through LC−MS/MS analysis. The field trials showed a significant reduction in ECB severity (75%), fungal ear rot severity (68%), *Fusarium Liseola* section infection (46%), FBs (75%) and MON (79%) as a result of the insecticide application for all the years, while the DON content increased by 60%. On the other hand, a fungicide application alone or applied in plots protected by an insecticide was never effective for the fungal symptoms, infection or mycotoxin content. The results confirm that a correct insecticide application to control ECB damage is the most effective agrochemical solution for the control of fungal ear rot, FBs and MON.

## 1. Introduction

Maize (*Zea mays* L.) is the world’s most important grain crop, in terms of yield, with an annual production estimated at around 1115 million tons [1]. Although most maize grain is used for animal feeds, this cereal is the basis of the diet of most people living in Latin America, Asia and Africa, and the consumption of this food crop is growing in developed countries as an ingredient for breakfast foods, dietetic products, baby foods, and as the main ingredient of many gluten−free formulations, including snacks, baked products and pasta [2]. In addition, after an industrial process related to the extraction of starch, maize grain is used as a raw material for a wide variety of other food products, such as candies, carbonated beverages, sauces and cake and soup mixes [3]. However, compared to other cereals, maize is subject to infection by a wider variety of mycotoxin-producing fungi. The most frequent significant diseases in temperate regions, in terms of mycotoxin contamination and consequent economic impact, are *Fusarium* ear rot, with *F. verticillioides*, *F. proliferatum* and *F. subglutinans* (all belonging to the *Liseola* section) as the main agents, *Gibberella* ear rot, caused by *F. graminearum* and *F. culmorum* (the *Discolor* section) and *Aspergillus* ear rot, in which the involved fungal species is *A. flavus* [4,5]. Four major mycotoxins, i.e., fumonisins (FBs, produced by the *Fusarium* spp. *Liseola* section), zearalenone and deoxynivalenol (DON, produced by the *Fusarium* spp. *Discolor* section) and aflatoxins (related to *Aspergillus* ear rot), are common to all maize-producing areas, although their relative importance and the ability to control them within the regulatory limits depend to a great extent on the environmental conditions [6]. Regulatory limits for these mycotoxins in maize have been established in the European Union [7,8], the USA [9] and other countries. Moniliformin (MON) is a mycotoxin that is frequently associated with FBs in maize grains [10]. *F. proliferatum*, which is also an FB producer, and *F. subglutinans,* are the main producers in temperate growing areas of this emerging mycotoxin [6,11]. Furthermore, MON could also be produced by many species of the *F. tricinctum* species complex, including *F. avenaceum* also in maize [12]. Still no regulatory limits have been established for MON, in spite of the fact that this compound has shown a high acute toxicity in rats [13]. Managing the risks of mycotoxin contamination requires an integrated approach that mainly focuses on the design, prior to the sowing, of cropping systems that are able to prevent the development of fungal species in the field [14]. The MycoKey maize working group has recently highlighted the importance of genetic resistance and insect control as the key factors to manage the mycotoxin contamination risk in maize [15]. It is in particular well known that the European Corn Borer (ECB), *Ostrinia nubilalis* (Hübner), promotes *Fusarium Liseola* section infection [10] in European maize growing areas, and the control of this pest, directly and indirectly, is one of the best strategies employed to minimize FB and MON contamination [6,16]. Since any genetic control involving Genetically Modified Organism (GMO) *Bt* technology is not allowed in several European countries, the control of ECB is achieved through insecticide applications during maize ripening using specific self-propelled ground sprayers [16,17,18]. The indirect control of ECB is related to such crop practices as the choice of the sowing date and the precocity of hybrids that are able to prevent ear injuries. In addition, several other agronomic practices, such as water management, fertilization and planting density, all of which are aimed at reducing crop stress, are known to play a secondary but substantial role in minimizing the mycotoxin content [15]. However, considering the health risk and economic losses of mycotoxin contamination, new control solutions are required to integrate the available preventive agronomic practices with the aim of reducing the risk of contamination. The application of synthetic fungicides is a well-known strategy to minimize the mycotoxin content in winter cereals [19] and, since 2009, the use of prothioconazole and tebuconazole has been approved for fungal ear rot control in maize in Italy. Although the ability of several fungicide active ingredients (a.i.) to control the *Fusarium* of both the *Liseola* and *Discolor* sections has been demonstrated under in vitro conditions [20], only a few studies have investigated the effect of fungicide treatments on mycotoxin occurrence in maize fields [17,18,21,22,23]. Moreover, these field studies compared just one possible fungicide application timing (at the flowering or at the milk stage, in combination with an insecticide treatment), and they have reported contrasting results. The application of fungicides to maize was also judged by the researchers involved in the MycoKey maize working group as being a less promising practice for FBs and aflatoxin, with a slightly higher value for only DON and zearalenone [15]. Furthermore, in a previous study, it was demonstrated that the timing of a fungicide application plays a key role in controlling maize foliar disease [24], as also recently confirmed in the study carried out against *Gibberella* ear rot by Limay-Rios and Schaafsma [21]. Considering the different ecologies and pathways of the fungal species involved in *Fusarium* ear rot, and their capacity to develop in all the ripening growth stages (GS), the research on the potential role of the direct control against FB, MON and DON producers requires field research that is able to cover several fungicide application timings, from maize flowering to the end of ripening. The aim of the current study has been to investigate the possibility of applying a direct control strategy to control the mycotoxins produced by *Fusarium* spp. through the application of synthetic fungicides as an additional solution in order to minimize the content of such mycotoxins in maize grain used for the food supply chain.

## 2. Results

### 2.1. Metereological Trends

The recorded rainfalls and the temperatures (expressed as growing degree days, GDDs) emphasized different trends between the three growing seasons (Table 1).

The year 2010 was characterized by cooler weather, with the lowest GDDs and the highest rainfall occurring during the growing season, although the rainfall events were more concentrated in May and June. Conversely, 2011 and 2012 had drier conditions, particularly during ripening. In 2011, the rainfall was more evenly distributed throughout the growing season compared to 2012, during which fewer precipitations occurred in the summer months (June–August).

### 2.2. Grain Yield

The insecticide application on average increased the grain yield significantly, that is, by 4% (Table 2). However, none of the fungicide timings influenced crop productivity to any great extent. The grain yield was highest in 2011, which was the year with the lowest number of injuries caused by ECB, followed by 2012 and 2010. The interaction between year and agrochemical treatment (insecticide and fungicide) was never significant.

### 2.3. ECB and Fungal Ear Rot Symptoms and Fungal Infection

**Figure 1:**
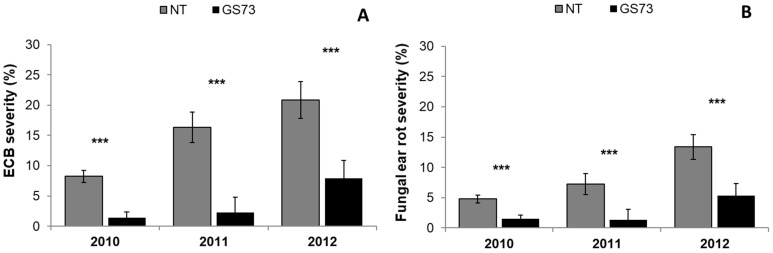
Effect of the insecticide application at the early milk growth stage (GS73) on European Corn Borer (ECB) severity (**A**) and fungal ear rot severity (**B**). The field experiments were carried out in 2010, 2011 and 2012 in northwest Italy. Bars (mean ± SEM; standard error of mean) with asterisks are significantly different: *** *p*-value < 0.001.

The pyrethroid insecticide application led to a significant reduction in ECB incidence (−62%) and severity (−75%), compared to the untreated control, and also resulted in a lower incidence (−54%) and severity (−68%) of fungal ear rot (Table 2). The fungicide application did not affect the symptoms caused by ECB or by fungal disease on the maize ears. The year 2011 on average showed less damage caused by ECB, followed by 2010 and 2012. The fungal ear rot incidence and severity followed the same trend over the growing seasons, thus confirming the importance of ECB as a pathway of the fungal species agent of ear rot. The interaction between insecticide and fungicide or fungicide and year was never significant. However, a significant interaction between insecticide treatment and year was reported for both ECB and the fungal ear rot symptoms: the effect of the insecticide significantly reduced ECB severity each year (*p*-value < 0.001; Figure 1), although the observed reduction was more marked in 2011 (−86%) and 2010 (−83%) than in 2012 (−62%). Similarly, the capacity of the insecticide application to minimize fungal ear rot severity was higher in 2011 (−81%) than in 2010 (−69%) or in 2012 (−60%).

**Figure 2:**
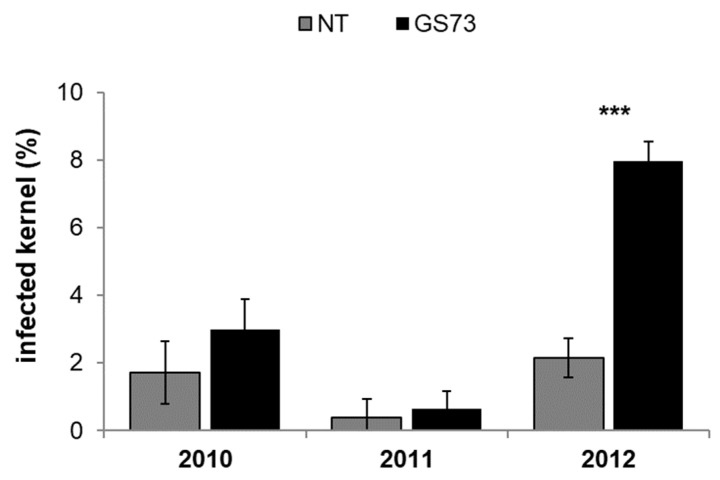
Effect of the insecticide application at the early milk growth stage (GS73) on the percentage of maize kernels infected by the *Fusarium* spp. *Discolor* section. The field experiments were carried out in 2010, 2011 and 2012 in northwest Italy. Bars (mean ± SEM; standard error of mean) with asterisks are significantly different: *** *p*-value < 0.001.

The insecticide showed significant but contrasting effects on the occurrence of the fungal species: the pyrethroid application significantly reduced the infection of *Fusarium* spp. section *Liseola* at the early milk stage, which in turn led to a significant increase in the species belonging to the *Discolor* section (Table 3). Conversely, the fungicide did not affect the incidence of kernels infected by either of the *Fusarium* section species. In accordance with the symptoms recorded on the ears at harvest, the year 2012 had the highest infection of both of the *Fusarium* section species. The interaction between insecticide and year for the *Fusarium* spp. *Discolor* section was significant: the application of this agrochemical treatment led to a significant increase (*p*-value < 0.001) in the number of infected kernels, albeit only for 2012 (Figure 2).

### 2.4. Mycotoxin Contamination

**Figure 3:**
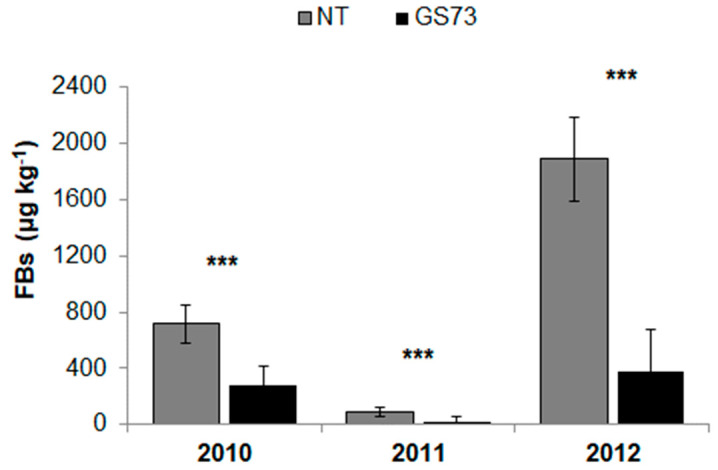
Effect of the insecticide application at the early milk growth stage (GS73) of maize on the contamination of fumonisins B_1_ + B_2_ (FBs). The field experiments were carried out in 2010, 2011 and 2012 in northwest Italy. Bars (mean ± SEM; standard error of mean) with asterisks are significantly different: *** *p*-value < 0.001.

The insecticide application significantly reduced the FB (−75%) and MON (−79%) contents, compared to the untreated control, while this application increased DON contamination by 60% (Table 4). On the other hand, the fungicide application timings did not affect the contamination of any of the considered mycotoxins. According to the fungal ear rot severity and infection, the contamination of all the *Fusarium* mycotoxins was the highest in 2012, followed by 2010 and 2011. The insecticide × fungicide and fungicide × year interactions were never significant, and a significant insecticide × year interaction was only observed for the FBs. The insecticide treatment significantly reduced the occurrence of FBs (*p*-value < 0.001), compared to the untreated control, in all the growing seasons (Figure 3), although the efficacy was higher in 2011 (−76%) and 2012 (−80%) than in 2010 (−60%).

## 3. Discussion

The results obtained under naturally infected field conditions and over three growing seasons confirmed that the control of the second generation larvae of ECB, by means of an insecticide application, was able to consistently reduce the severity of fungal ear rot and the contamination of FBs in grains intended for human consumption grown in non-Bt maize growing areas [6,22], and also resulted in a significant increase in grain yield. The effect of insecticides in minimizing the FB content was also consistent for the considered production situation of maize cultivated specifically for the food supply chain, in which all the preventive agronomic practices that are able to minimize the injuries of ECB and mycotoxin occurrence, such as early sowing and early harvest times and the careful management of plant density, fertilization and irrigation to avoid any plant stress, had already been applied [14]. The content of FBs in maize grain in the 2011 experiment was almost always below the limit for baby foods currently in force in the European Union (200 µg kg^−1^; [8]), while 46% of the samples complied with this limit in 2010 and 2012, albeit only when the insecticide was applied. As reported in previous studies, the optimum timing of pyrethroid insecticides to obtain the lowest FB contamination, as a consequence of the development of ECB injuries on maize ears, is between the beginning of a consistent adult flight activity and the flight peak, which generally occurs at the early milk GS in temperate growing areas and for maize planted early in the season [16]. Increasing the number of pyrethroid insecticide applications is not only not cost-effective, but it could affect non-target biota, especially the ECB’s natural enemies, leading to an increase in other pests, such as the *Tetranichus urticae* mite or aphids [25].

Furthermore, the control of ECB injuries also results in a significant reduction of other mycotoxins produced by *Fusarium* spp. of the *Liseola* section, such as MON [6]. In temperate areas, *F. verticillioides* and *F. proliferatum* infection is linked to ECB larvae feeding more than other *Fusarium* species, in particular those belonging to the *Discolor* section [16,26]; thus, it has been stated, in the scientific literature, that the application of an insecticide does not generally reduce the DON or ZEA contents [6,14], as has also been reported for the cultivation of *Bt* maize [27,28]. Furthermore, as also stated previously by Scarpino et al. [6] and Blandino et al. [14], the control of ECB injuries through the application of an insecticide could lead to a significant increase in DON production by the *Fusarium* spp. *Discolor* section. In the previous studies, the rise in the DON content as a consequence of an insecticide treatment was only reported when the environmental and agronomic conditions (full maturity hybrids, late planting time and consequent ripening) led to an overall high DON contamination as a consequence of a “flora inversion” phenomenon [17]: the control of ECB is able to modify the relative competitiveness of the *Fusarium* species during maize ripening by favoring the *Fusarium* spp. *Discolor* section, as a consequence of a lower occurrence of the *Liseola* section. In the present study, the flora inversion phenomenon was confirmed as both a fungal infection in maize grain at harvest and as the final content of FBs and DON, even in production situations with an overall low mycotoxin contamination level.

On the other hand, the field application of a fungicide, even when considering very comprehensive application timings within the maize crop cycle, that is, from silking to physiological maturity, over a period of approximately 60 days, did not significantly affect the fungal ear rot, fungal infection or mycotoxin contamination for either of the mycotoxins produced by the *Liseola* and *Discolor* sections. In vitro studies generally agree on a good efficacy of demethylation inhibitor (DMI) fungicides in controlling different *Fusarium* species. Masiello et al. [20] demonstrated that DMI fungicides, and prothioconazole in particular, are the most effective molecules against several strains of *F. graminearum*, *F. verticillioides*, *F. proliferatum* and *A. flavus*. Scaglioni et al. [22] and Li et al. [29] highlighted that tebuconazole and its enantiomers clearly inhibit *F. verticillioides* growth and could also reduce FB production, although the latter study reported that the effect could differ according to the environmental factors, such as the temperature and the water activity. Furthermore, some in vitro studies have highlighted that the use of fungicides could stimulate mycotoxin production in both the *Fusarium Liseola* section [30] and the *Discolor* section [31].

The chemical control of *Fusarium* species is still a key tool for the control of *Fusarium* head blight (FHB) in small cereals, and in particular in bread and durum wheat field programmes, and this control is achieved by spraying fungicides at flowering in order to avoid ear fungal infections [19]. DMI fungicides with triazole or triazolinthione active ingredients, and particularly prothioconazole, have shown a high efficacy in controlling FHB, and in minimizing both DON, produced by *F. graminearum* and *F. culmorum* [32], and other emerging mycotoxins, such as MON and enniatins, which are mainly produced in wheat by *F. avenaceum* [33]. Conversely, although some commercial formulations are currently available for maize, the application of a fungicide to control *Fusarium* ear rot is still not widely used, and the majority of studies have not demonstrated, in the field, the efficacy of synthetic fungicides on the reduction of mycotoxins associated with this disease. Andriolli et al. [34] reported that prothioconazole reduced ear rot caused by an artificial *F. meridionale* inoculation by 52% in Brazil when applied at silk emergence, while in the USA (Indiana), although prothioconazole, azoxystrobin + propiconazole and pyraclostrobin applied at maize silking were sometimes able to reduce disease severity when *F. graminearum* was artificially infected, fungicides never reduced DON contamination [35,36]. The lack of effectiveness of fungicides in minimizing DON and zearalenone was also reported in France by Folcher et al. [17] after applying tebuconazole at tassel emission. Limay-Rios and Schaafsma [21] recorded high efficacy for the application of a prothioconazole fungicide at silking (GS65) in controlling DON (−59%) and zearalenone (−57%) in Ontario. In this experiment, good control of mycotoxins produced by the *Discolor* section of the *Fusarium* genus was also recorded for the fungicide spray from the stage at which silk was completely emerged (GS63) to the silk browning stage (GS69), while no reduction of any mycotoxins was found after silk senescence (GS75, the milk stage). Limay-Rios and Schaafsma [21] set up a field experiment, which involved planting maize hybrids susceptible to silk *F. graminearum* infection late in the season, to encourage high natural infection. Furthermore, although FBs, MON and other emerging mycotoxins such as beauvericin and enniatins were only found in small quantities, they were not affected by the fungicide application. The same research group has recently reported that pydiflumetofen, a carboxamide active ingredient, sprayed at silking (GS65) reduced the DON content by 50%, and showed a similar efficacy to that of prothioconazole [23]. Again in this study, the fungicide treatments did not affect the FB content in the grains. However, the efficacy of fungicide against the *Fusarium Liseola* section and FB content has rarely been observed. Masiello et al. [20] reported that prothioconazole, when applied at silking, was effective in reducing *F. graminearum* and *F. proliferatum* infection, while it was less effective against *F. verticillioides*. In Argentina, metconazole and epoxiconazole applied at silking (GS65) did not affect the FB content for either natural infection or after inoculation with *F. verticillioides* [37]. The application of a fungicide in a mixture with an insecticide at the maize silking stage did not significantly affect the FB contamination, compared to the application of insecticide alone [17,18,22]. Scaglioni et al. [22], using prothioconazole + tebuconazole, and Blandino et al. [24], using azoxystrobin + propiconazole, also recorded no effect on FBs when the applications were made at the milk stage, and Small et al. [38], who double applied azoxystrobin + difeconazole at the stem elongation and blister stages, also reported no effect. On the other hand, in South Africa, the double application of the quinone outside inhibitor (QoI) and a DMI mixture at the pre-tassel stage and at silking instead resulted in a significant increase in the FB content in kernels at harvest [39]. Only in De Curtis et al. [40] did fungicide treatments applied in combination with insecticide lead to an additional reduction of FB, compared to an insecticide alone, but the experiment was carried out in a growing area in which maize cultivation is infrequent and considering a double fungicide application at the silking (GS65) and at early dough (GS83) stages. As far as other mycotoxin-producing genera are concerned, a few recent field studies have highlighted the efficacy of a fungicide application at silking in reducing *Aspergillus flavus* and aflatoxins in maize kernels at harvest [41,42].

The different levels of efficacy of the fungicide applications reported in literature may be explained by considering the different pathways of mycotoxin-producing species. Silk penetration seems to be the most important infection pathway for the *Fusarium Discolor* section, while infection mainly occurs in the *Fusarium Liseola* section through injuries caused during ripening, mainly by Lepidopteran insects, such as ECB [11,26]. For this reason, a fungicide application at silking is expected to protect more from DON- and aflatoxin-producing species than from FB ones. Furthermore, all the previously reported infection pathways are possible in temperate areas, thus the infection through open wounds during ripening should not be excluded for *F. graminearum* or *A. flavus*. In studies in Ontario in which a fungicide was shown to lead to high efficacy [21,23], there was no evident insect damage on maize ears, in part because, according to Limay-Rios and Schaafsma [21], only *Bt* transgenic hybrids were cultivated. The occurrence of ECB injuries during maize ripening recorded in the present experiment, even in insecticide-protected plots, in addition to the moderate *Fusarium Discolor* section infection, could support the lack of control of DON when a fungicide is applied at maize silking. On the other hand, the absence of fungicide control against FBs and MON could be related to a series of factors which differentiate the profitability of applying fungicide to maize in comparison to wheat. First, the long ripening period of maize in temperate growing areas (60–120 days from silking to harvest) makes different timings and pathway infections possible for FB producers, which may not be controlled by a single fungicide application. As previously reported, *F. verticillioides* and *F. proliferatum* could infect maize kernels at both flowering, through silks, or during ripening through kernel damage caused by insects. ECB larva activity may occur during all the ripening of GS in north Italy, as a consequence of the overlap of the second and third adult flights [16,25]. Thus, the absence of a precise and defined infection event makes it more difficult to identify the best application timing of a fungicide that would be able to protect the plants from fungal infection and development. Moreover, unlike wheat, which has exposed heads, maize ears are covered by the husk and located under several leaves, which results in a higher plant biomass. Anderson et al. [35] forwarded similar considerations, and highlighted that these fungicides only penetrate locally, without being fully systemic. The overcoming of these constraints would probably require the application of higher dosages of a.i. than those applied to small cereals, while the current commercial fungicide application rate is approximately the same for maize and wheat.

The application of prothioconazole + tebuconazole did not result in any yield benefits for any of the considered timings, thus confirming the results of Limay-Rios and Schaafsma [21] and Eli et al. [23]. By applying a QoI and DMI fungicide mixture, selected for the control of foliar disease and physiological benefits (azoxystrobin + propiconazole), Blandino et al. [24] reported a significant grain yield increase for an application between the nine leaf (GS19) and silking (GS65) stages, with the greatest effect at the stem elongation stage (GS35), while no productive benefit was found for an application at the milk stage (GS75). Thus, the profitability of a fungicide application, in terms of grain yield, may mainly be related to the preservation and the enhancement of the crop stay green, which can be better ensured through the application of a mixture of DMI and QoI or carboxamide a.i.

In conclusion, this study underlines the difficulties involved in applying a direct strategy to control and minimize the mycotoxin content in maize cultivated in temperate growing areas, where different *Fusarium* species, with several infection pathways, coexist. On the other hand, the application of fungicides at the maize silk stage could be more profitable in controlling DON and aflatoxins in production areas associated with infection mechanisms at such a stage. Thus, in growing areas where the cultivation of *Bt* maize is not allowed, the design of a cropping system that is able to prevent the overall development of ear diseases, integrated with the effective control of insect vectors through the use of a chemical insecticide, still remains the most effective strategy to minimize the risk of mycotoxin contamination in maize for the food supply chain.

## 4. Materials and Methods

### 4.1. Experimental Design

The effect of the application of insecticide and fungicide on the control of *Fusarium* infection and mycotoxin contamination in maize was studied over the 2010–2012 period in Cardè, northwest Italy (44°44′ N, 07°28′ E; altitude 258 m), in a deep and fertile sandy soil, *Typic Eutrochrepts* (USDA classification). The rainfall and temperature data were recorded daily from a weather station located next to the experimental field throughout the entire investigated period.

**Figure 4:**
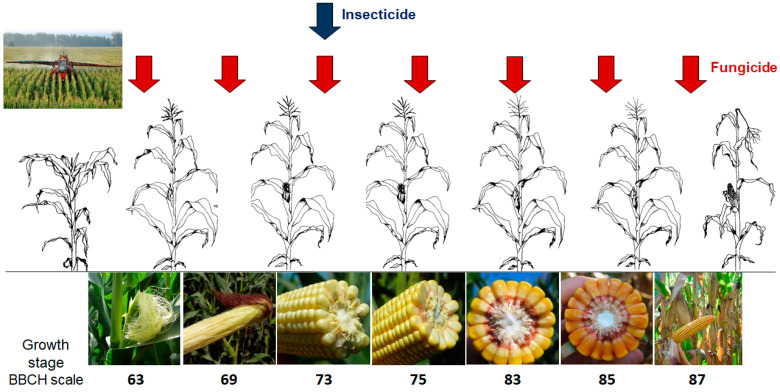
Scheduling of the different application timings of the fungicide and the single insecticide application compared in the field experiment, according to the BBCH growth stage of maize [43].

Different agrochemical foliar treatments were compared each year under naturally infected field conditions, considering a factorial combination of:

- Seven fungicide application timings, which were compared with an untreated control (NT). The fungicide treatments were applied once at approximately 10-day intervals, starting from maize flowering (GS63), until physiological maturity (GS87), at the GS reported in Figure 4;
- An insecticide treatment, which was applied at the early milk stage (GS73) to minimize the ear injuries caused by ECB activity, and compared with an untreated control (NT).

The sowing and harvest dates and the insecticide and fungicide application dates are reported in Table 5 for each year.

The treatments were randomly assigned to experimental units using a split-plot design, with the insecticide application as the main-plot treatment and the fungicide application as the sub-plot treatment. Each treatment combination was replicated three times. Each plot consisted of 25 m long rows spaced 0.75 m apart and separated by two untreated buffer rows on either side. The plot alleys, orthogonal to the maize rows, were 1 m wide.

The applied fungicide was a mixture of DMI prothioconazole + tebuconazole (Prosaro^®^, formulation: emulsifiable concentrate formulation, Bayer, Italy), and 0.125 kg of each active ingredient was applied (a.i.)/ha. The used insecticide was pyrethroid alpha-cypermethrin (Contest^®^, formulation: water dispersible granules, BASF, Cesano Maderno, Italy) applied at 0.044 kg of a.i. ha^−1^. The ECB flight activity was monitored by means of a cone trap placed outside the experimental plots, baited with sex pheromone (E:Z = 97:3) to attract males and with phenylacetaldehyde (PAA) for females. The sex pheromones and PAA dispenser were replaced every 15 and 30 days, respectively. Adults were removed from the trap and counted every 1−2 days. The insecticide was only applied at the milk stage (GS73), after the presence of the ECB flight peak had been observed.

Both the fungicide and insecticide treatments were applied by means of a self-propelled ground sprayer (GT3500, Grim^®^ srl, Jesi, Italy) according to the procedure reported in Scarpino et al. (2018) [6]. Studies were carried out each year on the Pioneer P1543 commercial dent corn hybrid (FAO rating 600; 130 days relative to maturity). A conventional agronomic technique for maize intended for the food supply chain was adopted for the field experiments in all of the growing seasons [6].

### 4.2. Grain Yield

Ears were collected, by hand, at harvest maturity from 4.5 m^2^ in the two central rows of each plot to quantify the grain yield and to obtain a representative sample for mycotoxin analysis. All the collected ears were shelled using a mechanical sheller. The kernels from each plot were mixed thoroughly to obtain a uniform sample, and grain moisture was analyzed using a Dickey−John GAC2100 grain analyzer (Auburn, IL, USA). The grain yields were adjusted to a 14% moisture content. A 5 kg sub-sample was taken for the mycotoxin analyses and dried at 60 °C for 72 h, in order to reduce the kernel moisture content to 10% before the milling operation.

### 4.3. ECB and Fungal Ear Rot Symptoms and Fungal Infection

A sub-sample of 30 ears was used, after removing the husk, to evaluate ECB and fungal ear rot severity at harvest, calculated as the percentage of kernels per ear with symptoms according to the procedure reported by Blandino et al. (2009) [16].

The evaluation of the *Fusarium* spp infections was carried out using 100 kernels randomly sampled from each plot at harvest. The kernels were surface disinfested for 3 min in a 2% solution of sodium hypochlorite, then rinsed 3 times with sterile water. The kernels were placed in Petri dishes containing dicloran cloramfenicol peptone (DCPA) and incubated at 20 °C. The *Fusarium* colonies were identified as belonging to the *Liseola* or *Discolor* sections after 7 to 10 days through colony and conidial morphology, as reported by Nelson et al. [44].

### 4.4. Mycotoxin Analysis

The FB, MON and DON quantifications were performed by applying three different methods as reported in Scaglioni et al. [22], Scarpino et al. [45] and Sovrani et al. [46].

Briefly, maize grain samples (5 kg for each plot) were all ground using a ZM 200 Ultra Centrifugal Mill, (Retsch GmbH, Haan, Germany) fitted with a 1 mm aperture sieve and the resulting whole meal, after a careful mixing operation, was used directly for the extraction. An aliquot of maize whole meal, for each mycotoxin analyzed, was extracted by mechanical shaking at 100 rpm (shaker mod. M102−OS, MPM Instruments, Milan, Italy), with methanol/water (80:20, *v*/*v*) for the FB extraction, with acetonitrile/water (84:16, *v*/*v*) for the MON extraction and with water for 30 min for the DON extraction [22,45,46]. The extracts were filtered through Whatman^®^ no. 1 filters (Brentford, UK) and subjected to clean-up and purification in different ways depending on the mycotoxin analyzed [22,45,46].

The purified extracts were transferred to HPLC vials and analyzed by means of LC−MS/MS analysis carried out on a Varian 310 triple quadrupole mass spectrometer (Agilent, Italy) equipped with an electrospray ionization ESI source, a 212 LC pump, a ProStar 410 AutoSampler and dedicated software. The chromatographic and mass spectrometric parameters of the investigated analytes were described in detail by Scaglioni et al. [22], Scarpino et al. [45] and Sovrani et al. [46].

### 4.5. Statistics

The Kolmogorov–Smirnov normality test and the Levene test were carried out to verify the normal distribution and homogeneity of variances, respectively. Analysis of the variance (ANOVA) was performed for the grain yield, ECB incidence and severity, fungal ear rot incidence and severity, fungal infection and mycotoxin content, with the insecticide application, the fungicide treatment timing and the year as independent factors. Multiple comparison tests were performed on the treatment means using the Ryan−Einot−Gabriel−Welsh F (REGW−F) test. For those parameters where the interaction between insecticide application and year was significant, ANOVA was performed separately for each year, with the insecticide treatment as an independent factor. Statistical data analysis was carried out with the SPSS software package, version 24.0.

## Figures and Tables

**Table 1:** Total rainfall, rainy days and growing degree days (GDDs) at the research site.

Year	Month	Rainfall	Rainy Days	GDDs ^1^
		mm	n°	Σ °C−day
2010	March	66	8	54
	April	59	8	143
	May	202	10	205
	June	146	8	313
	July	10	1	439
	August	59	5	345
	September	55	4	235
	October	119	5	114
	April–September	530	36	1680
	Flowering−Harvest	158	11	867
2011	March	211	11	68
	April	53	5	198
	May	42	5	269
	June	157	11	303
	July	71	7	344
	August	17	2	411
	September	64	5	336
	October	22	2	169
	April–September	405	35	1861
	Flowering−Harvest	152	14	1025
2012	March	26	2	148
	April	155	11	120
	May	96	7	236
	June	48	6	365
	July	47	7	404
	August	20	3	423
	September	77	8	249
	October	112	5	148
	April–September	444	42	1797
	Flowering−Harvest	128	17	1065

Data obtained from the Regione Piemonte agrometeorological service. ^1^ Accumulated growing degree days for each experiment using a 10 °C base value.

**Table 2:** Effect of the insecticide application and the timing of the fungicide application on the grain yield, incidence and severity of European Corn Borer (ECB) and fungal ear rot; field experiments carried out in northwest Italy in the 2010−2012 period.

Factor	Source of	Grain		ECB		ECB		Ear rot		Ear rot	
	Variation	Yield		Incidence ^1^		Severity ^2^		Incidence ^3^		Severity ^4^	
		(t ha^−1^)		(%)		(%)		(%)		(%)	
Insecticide (I)	NT	15.0	b	65.2	a	15.1	a	65.6	a	8.4	a
	GS73	15.6	a	25.0	b	3.9	b	30.3	b	2.7	b
	*p*-value	0.006		<0.001		<0.001		<0.001		<0.001	
	SEM	1.6		13.2		5.2		13.8		3.5	
Fungicide (F)	NT	15.2	a	48.8	a	10.5	a	50.1	a	5.9	a
	GS63	15.6	a	47.7	a	10.2	a	50.9	a	6.0	a
	GS69	15.4	a	44.5	a	9.1	a	45.8	a	5.0	a
	GS73	15.3	a	43.1	a	8.7	a	45.2	a	5.5	a
	GS75	15.3	a	44.4	a	9.7	a	47.5	a	6.1	a
	GS83	15.0	a	43.1	a	8.6	a	47.0	a	5.2	a
	GS85	15.2	a	44.0	a	9.0	a	48.8	a	5.1	a
	GS87	15.7	a	45.2	a	10.3	a	48.1	a	5.9	a
	*p*-value	0.730		0.690		0.779		0.784		0.893	
	SEM	3.0		24.7		9.7		25.8		6.6	
Year (Y)	2010	14.3	c	44.1	b	9.3	b	50.9	b	4.3	b
	2011	16.4	a	20.3	c	4.8	c	21.6	c	3.1	c
	2012	15.4	b	70.9	a	14.4	a	71.3	a	9.3	a
	*p*-value	<0.001		<0.001		<0.001		<0.001		<0.001	
	SEM	2.0		16.2		6.4		16.9		4.3	
I × F	*p*-value	0.915		0.964		0.951		0.971		0.425	
I × Y	*p*-value	0.534		<0.001		<0.001		0.001		<0.001	
F × Y	*p*-value	0.065		0.979		0.769		0.960		0.904	
I × F × Y	*p*-value	0.929		0.895		0.535		0.852		0.195	

Means followed by different letters are significantly different (the level of significance of the *p*-value is reported in the table), according to the REGW-F test. The reported data are the average of 72 replications (3 years × 8 fungicide treatments × 3 repetitions) for the insecticide treatments, and 18 replications (3 years × 2 insecticide treatments × 3 repetitions) for the fungicide treatments, while the data for each year are the average of 48 replications (2 insecticide × 8 fungicide treatments × 3 repetitions). SEM = standard error of the means. ^1^ ECB incidence was calculated as the percentage of ears with symptoms, considering 3 replications of 30 ears each. ^2^ ECB severity was calculated as the mean percentage of kernels with symptoms per ear, considering 3 replications of 30 ears each. ^3^ Fungal ear rot incidence was calculated as the percentage of ears with symptoms, considering 3 replications of 30 ears each.^4^ Fungal ear rot severity was calculated as the mean percentage of kernels with symptoms per ear, considering 3 replications of 30 ears each.

**Table 3:** Effect of the insecticide application and the timing of the fungicide application on the percentage of maize kernels infected by the *Fusarium* spp. *Liseola* and *Discolor* sections; field experiments carried out in northwest Italy in the 2010−2012 period.

Factor	Source of	*Fusarium* spp. Section (%)			
	Variation	*Liseola*		*Discolor*	
Insecticide (I)	NT	63.7	a	1.4	b
	GS73	34.2	b	3.9	a
	*p*-value	<0.001		0.004	
	SEM	10.8		4.0	
Fungicide (F)	NT	61.7	a	2.2	a
	GS63	44.4	a	3.4	a
	GS69	36.2	a	3.3	a
	GS73	47.8	a	2.2	a
	GS75	49.6	a	3.0	a
	GS83	45.1	a	1.0	a
	GS85	48.1	a	2.2	a
	GS87	58.9	a	3.8	a
	*p*-value	0.168		0.749	
	SEM	20.2		7.5	
Year (Y)	2010	41.1	b	2.3	b
	2011	34.9	b	0.5	b
	2012	71.0	a	5.1	a
	*p*-value	<0.001		<0.001	
	SEM	13.2		4.9	
I × F	*p*-value	0.674		0.546	
I × Y	*p*-value	0.082		0.018	
F × Y	*p*-value	0.696		0.485	
I × F × Y	*p*-value	0.442		0.295	

Means followed by different letters are significantly different (the level of significance of the *p*-value is reported in the table), according to the REGW-F test. SEM = standard error of the means. The reported data are the average of 72 replications (3 years × 8 fungicide treatments × 3 repetitions) for insecticide treatments, and 18 replications (3 years × 2 insecticide treatments × 3 repetitions) for fungicide treatments, while the data for each year are the average of 48 replications (2 insecticide × 8 fungicide treatments × 3 repetitions).

**Table 4:** Effect of the insecticide application and the fungicide application timing on the contamination of fumonisins B_1_ + B_2_ (FBs), moniliformin (MON) and deoxynivalenol (DON); field experiments carried out in northwest Italy in the 2010−2012 period.

Factor	Source of	FBs			MON			DON		
	Variation	T		NT (µg kg^−1^)	T		NT (µg kg^−1^)	T		NT (µg kg^−1^)
Insecticide (I)	NT	6.0	a	898	5.2	a	300	4.3	b	128
	GS73	4.4	b	226	3.2	b	63	4.6	a	205
	*p*-value	<0.001			<0.001			0.041		
	SEM	1.1			1.3			1.0		
Fungicide (F)	NT	5.6	a	603	4.7	a	261	4.2	a	133
	GS63	5.5	a	715	4.3	a	151	4.2	a	113
	GS69	4.9	a	499	3.8	a	222	4.4	a	145
	GS73	5.2	a	432	3.9	a	128	4.4	a	169
	GS75	4.9	a	447	4.2	a	190	4.9	a	268
	GS83	5.2	a	653	3.8	a	163	4.7	a	212
	GS85	5.2	a	680	4.6	a	195	4.4	a	129
	GS87	5.3	a	464	4.2	a	142	4.6	a	162
	*p*-value	0.212			0.080			0.204		
	SEM	2.0			2.5			1.9		
Year (Y)	2010	6.0	b	497	4.2	b	143	4.5	b	115
	2011	3.2	c	55	3.1	c	55	3.4	c	54
	2012	6.5	a	1133	5.2	a	347	5.5	a	331
	*p*-value	<0.001			<0.001			<0.001		
	SEM	1.3			1.7			1.3		
I × F	*p*-value	0.293			0.742			0.702		
I × Y	*p*-value	0.049			0.206			0.289		
F × Y	*p*-value	0.249			0.877			0.546		
I × F × Y	*p*-value	0.198			0.999			0.508		

The reported mycotoxin contamination means are transformed (T; y’= ln (x + 1)) and not transformed (N) values. Means followed by different letters are significantly different (the level of significance of the *p*-value is reported in the table), according to the REGW-F test. SEM = standard error of the means. The reported data are the average of 72 replications (3 years × 8 fungicide treatments × 3 repetitions) for the insecticide treatments, and 18 replications (3 years × 2 insecticide treatments × 3 repetitions) for fungicide treatments, while the data for each year are the average of 48 replications (2 insecticide × 8 fungicide treatments × 3 repetitions).

**Table 5:** Dates of the fungicide and insecticide applications, sowing and harvesting of the trials carried out over the 2010–2012 period in northwest Italy.

Fungicide Treatment	Maize Growth Stage (GS) ^1^	2010	2011	2012
NT	Untreated control	-	-	-
GS63	Flowering: tips of stigmata visible	12th July	4th July	4th July
GS69	End of flowering: stigmata completely dry	22nd July	15th July	15th July
GS73	Early milk	29th July	25th July	26th July
GS75	Milk ripening, about 40% dry matter	5th August	3rd August	5th August
GS83	Early dough, about 45% dry matter	19th August	16th August	16th August
GS85	Dough stage, about 55% dry matter	27th August	26th August	28th August
GS87	Physiological maturity	7th September	5th September	9th September
**Other agronomic information**				
Insecticide application	Early milk	29th July	25th July	26th July
Sowing date		2nd April	9th April	28th March
Harvesting date		6th October	27th September	4th October

^1^ According to the BBCH growth scale [42]. See Figure 4 for a graphical representation.

## Data Availability

The data that support the findings of this study are available from the corresponding author, [M.B.], upon reasonable request.

## References

[1] Food and Agriculture Organization of the United Nations (2020). FAOSTAT Statistical Database. https://www.fao.org/faostat/en/#home.

[2] Pellegrini N., Agostoni C. (2015). Nutritional aspects of gluten-free products. J. Sci. Food Agric..

[3] Yadav V.K., Supriya P., Chaudhary D., Kumar S., Langyan S. (2014). Value Addition in Maize. Maize: Nutrition Dynamics and Novel Uses.

[4] Munkvold G., Leslie J.F., Logrieco A.F. (2014). Crop management practices to minimize the risk of mycotoxins contamination in temperate-zone maize. Mycotoxin Reduction in Grain Chains.

[5] Battilani P., Toscano P., Van der Fels-Klerx H., Camardo Leggieri M., Brera C., Rortais A., Goumperis T., Robinson T. (2016). Aflatoxin B_1_ contamination in maize in Europe increases due to climate change. Sci. Rep..

[6] Scarpino V., Reyneri A., Sulyok M., Krska R., Blandino M. (2018). Impact of the insecticide application to maize cultivated in different environmental conditions on emerging mycotoxins. Field Crops Res..

[7] (2006). Commission regulation No. 1881/2006, of 10 December 2006 setting maximum levels for certain contaminants in food stuff. OJEU.

[8] (2006). Commission Regulation No 1126/2007 of 28 September 2007 amending Regulation (EC) No 1881/2006 setting maximum levels for certain contaminants in foodstuffs as regards *Fusarium* toxins in maize and maize products. OJEU.

[9] U.S. Food and Drug Administration (2001). Guidance for Industry: Fumonisin Levels in Human Foods and Animal Feeds; Final Guidance (6 June 2000; Revised 9 November 2001). https://www.fda.gov/regulatory-information/search-fda-guidance-documents/guidance-industry-fumonisin-levels-human-foods-and-animal-feeds.

[10] Scarpino V., Reyneri A., Vanara F., Scopel C., Causin R., Blandino M. (2015). Relationship between European Corn Borer injury, *Fusarium proliferatum* and *F. subglutinans* infection and moniliformin contamination in maize. Field Crops Res..

[11] Pfordt A., Ramos Romero L., Schiwek S., Karlovsky P., von Tiedemann A. (2020). Impact of Environmental Conditions and Agronomic Practices on the Prevalence of Fusarium Species Associated with Ear- and Stalk Rot in Maize. Pathogens.

[12] Ma N., Abdul Haseeb H., Xing F., Su Z., Shan L., Guo W. (2019). *Fusarium avenaceum*: A Toxigenic Pathogen Causing Ear Rot on Maize in Yunnan Province, China. Plant Dis..

[13] Jonsson M., Atosuo J., Jestoi M., Nathanail A.V., Kokkonen U.-M., Anttila M., Koivisto P., Lilius E.-M., Peltonen K. (2015). Repeated Dose 28-Day Oral Toxicity Study of Moniliformin in Rats. Toxicol. Lett..

[14] Blandino M., Reyneri A., Vanara F., Tamietti G., Pietri A. (2009). Influence of agricultural practices on *Fusarium* infection, fumonisin and deoxynivalenol contamination of maize kernels. World Mycotoxin J..

[15] Logrieco A., Battilani P., Camardo Leggieri M., Jiang Y., Haesaert G., Lanubile A., Mahuku G., Mesterházy A., Ortega-Beltran A., Pasti M. (2021). Perspectives on Global Mycotoxin Issues and Management from the MycoKey Maize Working Group. Plant Dis..

[16] Blandino M., Reyneri A., Vanara F., Pascale M., Haidukowski M., Campagna C. (2009). Management of fumonisin contamination in maize kernels through the timing of insecticide application against the European corn borer *Ostrinia nubilalis* Hübner. Food Addit. Contam. Part A.

[17] Folcher L., Jarry M., Weissenberger A., Gérault F., Eychenne N., Delos M., Regnault-Roger C. (2009). Comparative activity of agrochemical treatments on mycotoxin levels with regard to corn borers and *Fusarium* mycoflora in maize (*Zea mays* L.) fields. Crop Prot..

[18] Mazzoni E., Scandolara A., Giorni P., Pietri A., Battilani P. (2011). Field control of *Fusarium* ear rot, *Ostrinia nubilalis* (Hübner), and fumonisins in maize kernels. Pest Manag. Sci..

[19] Blandino M., Scarpino V., Sulyok M., Krska R., Reyneri A. (2017). Effect of agronomic programmes with different susceptibility to deoxynivalenol risk on emerging contamination in winter wheat. Eur. J. Agron..

[20] Masiello M., Somma S., Ghionna V., Logrieco A.F., Moretti A. (2019). In Vitro and in Field Response of Different Fungicides against *Aspergillus flavus* and *Fusarium* Species Causing Ear Rot Disease of Maize. Toxins.

[21] Limay-Rios V., Schaafsma A.W. (2018). Effect of Prothioconazole Application Timing on *Fusarium* Mycotoxin Content in Maize Grain. J. Agric. Food Chem..

[22] Scaglioni P., Blandino M., Scarpino V., Giordano D., Testa G., Badiale-Furlong E. (2018). Application of fungicides and microalgal phenolic extracts for the direct control of fumonisin contamination in maize. J. Agric. Food Chem..

[23] Eli K., Schaafsma A.W., Limay-Rios V., Hooker D.C. (2021). Effect of pydiflumetofen on Gibberella ear rot and Fusarium mycotoxin accumulation in maize grain. World Mycotoxin J..

[24] Blandino M., Galeazzi M., Savoia W., Reyneri A. (2012). Timing of azoxystrobin + propiconazole application on maize to control Northern Corn Leaf Blight and maximize grain yield. Field Crops Res..

[25] Saladini M., Blandino M., Reyneri A., Alma A. (2008). The impact of insecticide treatments on *Ostrinia nubilalis* (Hübner) (Lepidoptera: Crambidae) and their influence on the mycotoxin contamination of maize kernels. Pest Manag. Sci..

[26] Munkvold G.P. (2003). Epidemiology of *Fusarium* diseases and their mycotoxins in maize ears. Eur. J. Plant Path..

[27] Masoero F., Meschini M., Rossi F., Grandini A., Pietri A. (1999). Nutritive value, mycotoxin contamination and in vitro rumen fermentation of normal and genetically modified corn (Cry1A(B)) grown in northern Italy. Maydica.

[28] Folcher L., Delos M., Marengue E., Jarry M., Weissenberger A., Eychenne N., Regnault-Roger C. (2010). Lower mycotoxin levels in Bt maize grain. Agron. Sustain. Dev..

[29] Li N., Zhao J., Zhang R., Deng L., Li J., Gao Y., Liu C. (2018). Effect of Tebuconazole Enantiomers and Environmental Factors on Fumonisin Accumulation and FUM Gene Expression in *Fusarium verticillioides*. J. Agric. Food Chem..

[30] Miguel T.A., Bordini J.G., Saito G.H., Andrade C.G.T.J., Ono M.A., Hirooka E.Y., Vizoni E., Ono E.Y.S. (2015). Effect of fungicide on *Fusarium verticillioides* mycelia morphology and fumonisin B_1_ production. Braz. J. Microbiol..

[31] Doohan F.M., Weston G., Rezanoor H.N., Parry D.W. (1999). Development and use of a reverse transcription—PCR assay to study the expression of tri5 by *Fusarium* species “in vitro” and “in planta”. Appl. Environ. Microbiol..

[32] Paul P.A., Lipps P.E., Hershman D.E., McMullen M.P., Draper M.A., Madden L.V. (2008). Efficacy of triazole-based fungicides for Fusarium Head Blight and deoxynivalenol control in wheat: A multivariate meta-analysis. Phytopathology.

[33] Scarpino V., Reyneri A., Sulyok M., Krska R., Blandino M. (2015). Effect of fungicide application to control *Fusarium* head blight and *Fusarium* and *Alternaria* mycotoxins in winter wheat (*Triticum aestivum* L.). World Mycotoxin J..

[34] Andriolli C.F., Casa R.T., Kuhnem P.R., Kuhmen P.R., Bogo A., Luis Zancan R., Melo Reis E. (2016). Timing of fungicide application for the control of Gibberella ear rot of maize. Trop. Plant Pathol..

[35] Anderson N.R., Romero Luna M.P., Ravellette J.D., Wise K.A. (2017). Impact of Foliar Fungicides on Gibberella Ear Rot and Deoxynivalenol Levels in Indiana Corn. Plant Health Prog..

[36] Parker N.S., Anderson N.R., Richmond D.S., Long E.Y., Wise K.A., Krupke C.H. (2017). Larval western bean cutworm feeding damage encourages the development of Gibberella ear rot on field corn. Pest Manag. Sci..

[37] Abdala L.J., Gerde J.A., Gambin B.L., Borrás L. (2018). Fungicide Applications and Grain Dry Milling Quality in Late-Sown Maize. Crop Sci..

[38] Small I.M., Flett B.C., Marasas W.F.O., McLeod A., Viljoen A. (2012). Use of resistance elicitors to reduce *Fusarium* ear rot and fumonisin accumulation in maize. Crop Prot..

[39] Janse van Rensburg B., Mc Laren N.W., Schoeman A., Flett B.C. (2016). The effects of cultivar and prophylactic fungicide spray for leaf diseases on colonisation of maize ears by fumonisin producing *Fusarium* spp. and fumonisin synthesis in South Africa. Crop prot..

[40] De Curtis F., De Cicco V., Haidukowski M., Pascale M., Somma S., Moretti A. (2011). Effects of agrochemical treatments on the occurrence of Fusarium ear rot and fumonisin contamination of maize in Southern Italy. Field Crops Res..

[41] Ferrigo D., Mondin M., Scopel C., Dal Maso E., Stefenatti M., Raiola A., Causin R. (2019). Effects of a prothioconazole- and tebuconazole-based fungicide on *Aspergillus flavus* development under laboratory and field conditions. Eur. J. Plant Pathol..

[42] Lagogianni S., Tsitsigiannis D.I. (2018). Effective chemical management for prevention of aflatoxins in maize. Phytopathol. Mediterr..

[43] Lancashire P.D., Bleiholder H., Longelüddcke P., Stauss R., Van Den Boom T., Weber E., Witzenberger A. (1991). An uniform decimal code for growth stages of crops and weeds. Ann. Appl. Biol..

[44] Nelson P.E., Toussoun T.A., Marasas W.F.O. (1983). Fusarium Species: An Illustrated Manual for Identification.

[45] Scarpino V., Blandino M., Negre M., Reyneri A., Vanara F. (2013). Moniliformin analysis in maize samples from North-West Italy using multifunctional clean-up columns and the LC-MS/MS detection method. Food. Addit. Contam. Part A Chem. Anal. Control Expo. Risk Assess..

[46] Sovrani V., Blandino M., Scarpino V., Reyneri A., Coisson J.D., Travaglia F., Locatelli M., Bordiga M., Montella R., Arlorio M. (2012). Bioactive compound content, antioxidant activity, deoxynivalenol and heavy metal contamination of pearled wheat fractions. Food Chem..

